# Delivery-related risk factors for covert postpartum urinary retention after vaginal delivery

**DOI:** 10.1007/s00192-015-2768-8

**Published:** 2015-07-30

**Authors:** Femke E. M. Mulder, Katrien Oude Rengerink, Joris A. M. van der Post, Robert A. Hakvoort, Jan-Paul W. R. Roovers

**Affiliations:** Department of Obstetrics and Gynaecology, Academic Medical Centre, Meibergdreef 9, 1105 AZ Amsterdam, The Netherlands; Department of Obstetrics and Gynaecology, Spaarne Hospital, Hoofddorp, The Netherlands

**Keywords:** Postpartum period, Postpartum urinary retention, Incomplete voiding, Voiding dysfunction, Risk factors, Vaginal delivery

## Abstract

**Introduction and hypothesis:**

Postpartum urinary retention (PUR) is a common consequence of bladder dysfunction after vaginal delivery. Patients with covert PUR are able to void spontaneously but have a postvoid residual bladder volume (PVRV) of ≥150 mL. Incomplete bladder emptying may predispose to bladder dysfunction at a later stage of life. The aim of this cross-sectional study was to identify independent delivery-related risk factors for covert PUR after vaginal delivery in order to identify women with an increased risk of covert PUR.

**Methods:**

The PVRV of women who delivered vaginally was measured after the first spontaneous micturition with a portable bladder-scanning device. A PVRV of 150 mL or more was defined as covert PUR. Independent risk factors for covert PUR were identified in multivariate regression analysis.

**Results:**

Of 745 included women, 347 (47 %) were diagnosed with covert PUR (PVRV ≥150 mL), of whom 197 (26 %) had a PVRV ≥250 mL (75th percentile) and 50 (7 %) a PVRV ≥500 mL (95th percentile). In multivariate regression analysis, episiotomy (OR 1.7, 95 % CI 1.02 – 2.71), epidural analgesia (OR 2.08, 95 % CI 1.36 – 3.19) and birth weight (OR 1.03, 95 % CI 1.01 – 1.06) were independent risk factors for covert PUR. Opioid analgesia during labour (OR 3.19, 95 % CI 1.46 – 6.98), epidural analgesia (OR 3.54, 95 % CI 1.64 – 7.64) and episiotomy (OR 3.72, 95 % CI 1.71 – 8.08) were risk factors for PVRV ≥500 mL.

**Conclusions:**

Episiotomy, epidural analgesia and birth weight are risk factors for covert PUR. We suggest that the current cut-off values for covert PUR should be reevaluated when data on the clinical consequences of abnormal PVRV become available.

## Introduction

In the puerperium, postpartum urinary retention (PUR) is a common finding which gives an increased risk of persistent urinary retention [1–6]. Reported prevalences for overt (symptomatic) PUR range from 0.3 % to 4.7 %, i.e. the inability to void spontaneous within 6 h of vaginal delivery or removal of a catheter after a caesarean section [1, 7]. For covert (asymptomatic) PUR, defined as a postvoid residual volume (PVRV) of at least 150 mL after spontaneous micturition, prevalences of even up to 45 % have been reported [2].

Since Yip et al. proposed a distinction between overt and covert PUR in 1997 [1], many authors have adopted these definitions, which has led to a more consistent comparison between studies that deal with this common problem. The distinction between overt and covert PUR has clinical consequences. Women who are unable to micturate spontaneously within 6 h of delivery are categorized as having overt (symptomatic) urinary retention. Covert (asymptomatic) urinary retention is defined as the presence of a PVRV of more than 150 mL, detected by ultrasonography or by catheterization after spontaneous micturition.

Numerous studies have shown spontaneous recovery after several days to a normal PVRV in women with covert PUR [1, 5, 8, 9]. A recent systematic review on the adverse effects of PUR has shown that there is insufficient evidence to state that covert PUR is harmless [10]. However, it is known that over-distension of the bladder, even a single episode of over-distension, can lead to long-lasting voiding difficulties, recurrent urinary tract infections and, rarely, impaired renal function [11–13]. Sometimes long-term catheterization may be indicated when retention persists or irreversible damage to the urogenital tract has occurred. Possibly, screening for covert PUR might be indicated to limit these risks.

This cross-sectional study was performed to identify risk factors for covert PUR.

## Materials and methods

Between September 2010 and January 2013, data on the PVRV of women after vaginal delivery were collected in an academic hospital in The Netherlands. In this hospital, an average of 1,600 women per year give birth, with a caesarean section rate of 25 %, resulting in 1,200 vaginal deliveries each year. Women with an indication for prolonged catheterization because of their general condition (for example, severe pre-eclampsia or a retained placenta) were excluded, as well as women with a twin pregnancy. For women suffering severe fetomaternal pathology, eligibility was judged by the nurse who took care of the patient after the delivery. In women receiving epidural analgesia, the indwelling catheter was removed during the second stage of labour.

In participating women the first voided volume was measured. If micturition on a toilet was not possible, women were given the opportunity to void while showering. Within a maximum of 15 min after the first void the PVRV was measured with a portable noninvasive abdominal ultrasound device (Bladderscan® BVI 9400; Verathon Medical Europe, IJsselstein, The Netherlands). Nurses were trained in the appropriate use of the bladder-scanning device. The PVRV was recorded on the electronic patient chart as well as in a paper file. Potential clinical risk factors were identified based on literature [1, 14, 15] and were subsequently collected from (electronic) patient charts. The majority of the included factors are obligatory items on the patient chart, facilitating reliable documentation. In women diagnosed with covert PUR, PVRV was measured repeatedly until it was normal.

Clinical risk factors for the development of covert PUR were analysed using SPSS (IBM Statistics, version 20), with univariate regression analysis. Analysis was performed for a PVRV cut-off value of ≥150 mL, being the most common value in the literature. After identifying the 75th and 95th percentiles, analyses were also performed for the PVRV values related to these percentiles. Predictors with a *p* value <0.20 were included in a multivariate regression model. Associations between potential predictors and outcome are reported as odds ratios with 95 % confidence intervals. Since bladder scanning was part of standard postpartum care, no ethical approval was required.

## Results

Between September 2010 and January 2013, data were obtained for 930 women, and the PVRV in 745 (80 %) of these women could be used in the analysis. No documentation of the PVRV was available in 165 women, and 20 women were excluded because they had a twin pregnancy. The patient characteristics are shown in Table 1. The mean age of the women was 31 years, their median parity was 2, and 13 % (94/745) underwent an instrumental delivery (all instrumental deliveries were vacuum extractions).

**Table 1:** Baseline characteristics of the 745 included women

Characteristic	Value
Maternal age (years), mean (range)	31 (16 – 46)
BMI (kg/m^2^), median (range)	24 (16 – 64)
Parity, median (range)	1.8 (1 – 8)
Spontaneous vaginal delivery, *n* (%)	651 (87)
Instrumental delivery, *n* (%)	94 (13)
Epidural analgesia, *n* (%)	141 (19)
Opioid analgesia, *n* (%)	121 (16)
Episiotomy, *n* (%)	131 (18)

**Fig. 1:**
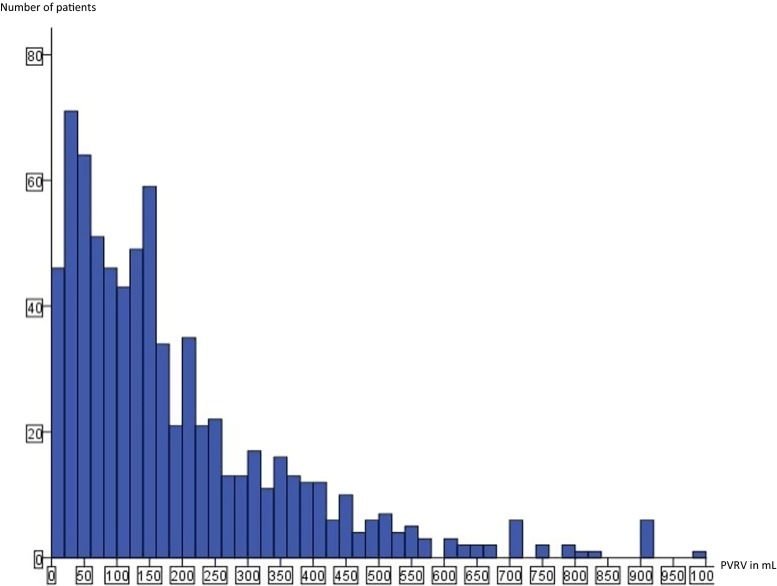
PVRV measurements

The first voided volume was not measured routinely and not when the first void took place during showering, andthus was measured in 439 of the 745 women. In these 439 women the median first voided volume was 320 mL (range of 30 – 1,900 mL). The median PVRV was 140 mL (0 – 1,000 mL), and the 75th and 95th percentiles were 250 mL and 540 mL, respectively (Fig. 1). For ease of interpretation and use in practice the values used in the regression model were median 150 mL (value often used in previous studies), 75th percentile 250 mL and 95th percentile 500 mL as outcomes. Of the 745 women, 347 (47 %) were diagnosed with covert PUR (PVRV ≥150 mL), of whom 197 (26 %) had a PVRV ≥250 mL (75th percentile) and 50 (7 %) a PVRV ≥500 mL (95th percentile).

Table 2 shows the univariable regression analyses, using the 150 mL, 250 mL and 500 mL cut-off values. Primiparity, duration of labour, duration of the second stage of labour, opioid analgesia (i.e. intramuscular morphine or intravenous remifentanil), epidural analgesia, instrumental delivery, birth weight, vaginal tears requiring suturing, episiotomy and augmentation were univariably associated with covert PUR (PVRV ≥150 mL).

**Table 2:** Univariable regression analysis

Clinical factor	Number of women	PVRV ≥150 mL		PVRV ≥250 mL		PVRV ≥500 mL	
		OR	95 % CI	OR	95 % CI	OR	95 % CI
Maternal age (per year)	745	1.01	0.99 – 1.04	1.03	0.98 – 1.03	1.03	0.97 – 1.08
BMI (per kg/m^2^)	625	0.99	0.96 – 1.01	0.98	0.95 – 1.01	1.00	0.95 – 1.05
Primiparous (yes/no)	745	1.63	1.22 – 2.18	1.46	1.05 – 2.03	2.56	1.39 – 4.73
Duration of labour (per minute)	724	1.00	1.00 – 1.00	1.00	1.00 – 1.00	1.00	1.00 – 1.00
Duration of second stage (per minute)	738	1.01	1.01 – 1.02	1.01	1.01 – 1.02	1.01	1.00 – 1.02
Augmentation (yes/no)	745	1.30	0.97 – 1.73	1.20	0.87 – 1.67	1.17	0.65 – 2.08
Opioid analgesia (yes/no)	745	1.39	0.93 – 2.08	2.04	1.32 – 3.14	3.59	1.72 – 7.50
Epidural (yes/no)	745	2.57	1.75 – 3.77	2.08	1.41 – 3.06	2.88	1.57 – 5.26
Instrumental delivery (yes/no)	745	2.49	1.58 – 3.92	1.89	1.20 – 2.97	3.34	1.75 – 6.41
Birth weight (per 100 g)	744	1.04	1.01 – 1.06	1.05	1.02 – 1.07	1.03	0.99 – 1.08
Vaginal tears (yes/no)	745	1.58	1.17 – 2.14	1.46	1.03 – 2.07	3.26	1.50 – 7.04
Episiotomy (yes/no)	745	2.39	1.61 – 3.53	2.83	1.91 – 4.19	5.07	2.81 – 9.17

After selection of possible risk factors through univariable regression, multivariate analysis revealed epidural analgesia, birth weight and episiotomy as independent risk factors for PVRV ≥150 mL (Table 3). For the PVRV cut-off value of ≥250 mL, opioid analgesia, birth weight, epidural analgesia and episiotomy were risk factors. For the PVRV cut-off value of ≥500 mL, opioid analgesia, epidural analgesia and episiotomy were significant risk factors.

**Table 3:** Multivariable regression analysis including factors with *p* < 0.20 in the univariable analysis (Table 2; maternal age, BMI and augmentation were not included)

Clinical factor	PVRV ≥150 mL		PVRV ≥250 mL		PVRV ≥500 mL	
	OR	95 % CI	OR	95 % CI	OR	95 % CI
Primiparous (yes/no)	1.26	0.83 – 1.81	1.13	0.75 – 1.70	1.60	0.77 – 3.33
Duration of labour (per minute)	1.00	1.00 – 1.00	1.00	1.00 – 1.00	1.00	1.00 – 1.00
Duration of second stage (per minute)	1.00	1.00 – 1.01	1.01	1.00 – 1.01	1.00	0.98 – 1.01
Opioid analgesia (yes/no)	1.18	0.77 – 1.81	1.86*	1.18 – 2.94	3.19*	1.46 – 6.98
Epidural analgesia ^(yes/no)^	2.08*	1.36 – 3.19	2.07*	1.32 – 3.26	3.54*	1.64 – 7.64
Instrumental delivery (yes/no)	1.35	0.78 – 2.34	0.85	0.48 – 1.49	1.15	0.52 – 2.52
Birth weight (per 100 g)	1.03*	1.01 – 1.06	1.04*	1.01 – 1.07	1.03	0.97 – 1.08
Vaginal tears (yes/no)	1.07	0.76 – 1.52	0.86	0.57 – 1.29	1.53	0.64 – 3.68
Episiotomy (yes/no)	1.67*	1.02 – 2.71	2.53*	1.53 – 4.20	3.72*	1.71 – 8.08

*P < 0.05

## Discussion

This multivariate regression analysis showed that episiotomy, epidural analgesia and birth weight are independent risk factors for covert PUR (PVRV ≥150 mL). The 75th percentile of measured PVRV in all women was 250 mL; for this cut-off value, opioid analgesia, birth weight, epidural analgesia and episiotomy were identified as independent risk factors. Our applied lower limit of 150 mL for the diagnosis of covert PUR is in line with previous reports [1, 16]. The use of this cut-off value facilitates comparison with other studies [1, 8, 14]. Although this was a large cross-sectional study with data from over 700 women on covert PUR after vaginal delivery, some potential limitations need discussing.

First, selection bias may have occurred as women with complicated deliveries (i.e. extreme premature deliveries, postpartum haemorrhage, severe pre-eclampsia) were not always screened for PVRV. However, we have no reason to believe that voiding mechanisms would be different in this (small) group of woman with severe pregnancy-related complications and therefore believe our results can be generalized. In order to confirm our hypothesis, we plan to perform an external validation.

Second, the use of a Bladderscan as a technique for measuring PVRV has often been discussed. The validation studies that have been performed suggest that with such devices reliable measurements can be obtained directly after birth, while no statistically significant differences have been found between abdominal measurements and catheterization [17–20]. In our department, a modern 3D model was used (Bladderscan BVI 9400) and the nursing staff was also extensively trained. Therefore, all measures were taken to achieve optimal reliability of measurements.

One of the largest studies concerning this subject is a recent study by Buchanan and Beckmann who found that primiparity and large perineal tears as well as caesarean section are independent predictors [8]. Although this was a large study it cannot be easily compared with ours due to a high percentage of women undergoing caesarean section and a relatively large number of women receiving regional anaesthesia. As no information was given in the previous study about catheter placement protocols, it is hard to compare the previous study with ours because we did not include women after caesarean section nor women who had received regional anaesthesia. However, the finding of perineal tears as a predictor is in line with the findings of our study and several others.

Because in our large cohort the median PVRV after the first void was 140 mL, the 75th percentile 250 mL and the 95th percentile 540 mL, we believe that the currently used definition proposed by Yip et al. should be reconsidered. While the classification of Yip et al. is based on the arbitrary PVRV of 150 mL in women who void at least 9 h after vaginal delivery, we feel that the definition of postpartum PUR should be related to the first postpartum void that occurs during the first hours after delivery.

Our study showed that opioid analgesia, epidural analgesia and episiotomy are risk factors for a PVRV that exceeds 500 mL. Although the exact pathophysiological background of PUR is still unclear, many hypotheses have been suggested, including anatomical changes [21], enlargement of bladder capacity [22] and hormonal changes during pregnancy [23]. Obviously, vaginal delivery is an anatomically and functionally traumatic event as it not only influences the anatomy and pelvic floor muscles [24] but also has effects on pudendal nerve conduction [25, 26] and possibly causes obstructive periurethral and vulval oedema. Our results showed that episiotomy, epidural analgesia and birth weight independently influence postpartum bladder function negatively. The (sutured) episiotomy as a predictor is likely to exert its effect through the development of pain and subsequent disturbance in bladder sensitivity and also central inhibition of bladder function [27–29].

It is rational to assume that the more extreme change in anatomy that occurs in primiparous women is different from the change that occurs in the more adapted pelvic floor of multiparous women. It is likely that the first situation results in more pain. From this mechanical point of view, it is also likely that the birth of neonates with a larger birth weight can cause more trauma, subsequently resulting in a more painful delivery with eventually an inhibitory effect on bladder function. Last, the finding of epidural analgesia as a predictor of abnormal PVRV as an expression of decreased bladder function was not surprising [7, 30] as it directly affects (bladder) sensitivity and contractility.

These hypotheses could provide an explanation as to how these clinical factors cause an abnormal PVRV. Although several authors have shown that PVRV often normalizes spontaneously [1, 2, 5, 14] data on long-term and adverse effects are still missing. A recent systematic review on adverse effects of PUR has shown that there is insufficient evidence to state that covert PUR harmless [10]. Future research should therefore also focus on clinical consequences and long-term adverse effects related to the occurrence of covert PUR.

## Conclusions

Episiotomy, epidural analgesia and birth weight are independent risk factors for covert PUR. We suggest that the current cut-off values for covert PUR should be reevaluated when data on the clinical consequences of abnormal PVRV become available.
